# Risk factors for SARS-CoV-2 among patients in the Oxford Royal College of General Practitioners Research and Surveillance Centre primary care network: a cross-sectional study

**DOI:** 10.1016/S1473-3099(20)30371-6

**Published:** 2020-09

**Authors:** Simon de Lusignan, Jienchi Dorward, Ana Correa, Nicholas Jones, Oluwafunmi Akinyemi, Gayatri Amirthalingam, Nick Andrews, Rachel Byford, Gavin Dabrera, Alex Elliot, Joanna Ellis, Filipa Ferreira, Jamie Lopez Bernal, Cecilia Okusi, Mary Ramsay, Julian Sherlock, Gillian Smith, John Williams, Gary Howsam, Maria Zambon, Mark Joy, F D Richard Hobbs

**Affiliations:** a Nuffield Department of Primary Care Health Sciences, University of Oxford, Oxford, UK; b Royal College of General Practitioners Research and Surveillance Centre, London, UK; c Centre for the AIDS Programme of Research in South Africa, University of KwaZulu–Natal, Durban, South Africa; d Institute for Global Health, University College London, London, UK; e Section of Clinical Medicine, University of Surrey, Guildford, UK; f Public Health England, London, UK

## Abstract

**Background:**

There are few primary care studies of the COVID-19 pandemic. We aimed to identify demographic and clinical risk factors for testing positive for severe acute respiratory syndrome coronavirus 2 (SARS-CoV-2) within the Oxford Royal College of General Practitioners (RCGP) Research and Surveillance Centre primary care network.

**Methods:**

We analysed routinely collected, pseudonymised data for patients in the RCGP Research and Surveillance Centre primary care sentinel network who were tested for SARS-CoV-2 between Jan 28 and April 4, 2020. We used multivariable logistic regression models with multiple imputation to identify risk factors for positive SARS-CoV-2 tests within this surveillance network.

**Findings:**

We identified 3802 SARS-CoV-2 test results, of which 587 were positive. In multivariable analysis, male sex was independently associated with testing positive for SARS-CoV-2 (296 [18·4%] of 1612 men *vs* 291 [13·3%] of 2190 women; adjusted odds ratio [OR] 1·55, 95% CI 1·27–1·89). Adults were at increased risk of testing positive for SARS-CoV-2 compared with children, and people aged 40–64 years were at greatest risk in the multivariable model (243 [18·5%] of 1316 adults aged 40–64 years *vs* 23 [4·6%] of 499 children; adjusted OR 5·36, 95% CI 3·28–8·76). Compared with white people, the adjusted odds of a positive test were greater in black people (388 [15·5%] of 2497 white people *vs* 36 [62·1%] of 58 black people; adjusted OR 4·75, 95% CI 2·65–8·51). People living in urban areas versus rural areas (476 [26·2%] of 1816 in urban areas *vs* 111 [5·6%] of 1986 in rural areas; adjusted OR 4·59, 95% CI 3·57–5·90) and in more deprived areas (197 [29·5%] of 668 in most deprived *vs* 143 [7·7%] of 1855 in least deprived; adjusted OR 2·03, 95% CI 1·51–2·71) were more likely to test positive. People with chronic kidney disease were more likely to test positive in the adjusted analysis (68 [32·9%] of 207 with chronic kidney disease *vs* 519 [14·4%] of 3595 without; adjusted OR 1·91, 95% CI 1·31–2·78), but there was no significant association with other chronic conditions in that analysis. We found increased odds of a positive test among people who are obese (142 [20·9%] of 680 people with obesity *vs* 171 [13·2%] of 1296 normal-weight people; adjusted OR 1·41, 95% CI 1·04–1·91). Notably, active smoking was linked with decreased odds of a positive test result (47 [11·4%] of 413 active smokers *vs* 201 [17·9%] of 1125 non-smokers; adjusted OR 0·49, 95% CI 0·34–0·71).

**Interpretation:**

A positive SARS-CoV-2 test result in this primary care cohort was associated with similar risk factors as observed for severe outcomes of COVID-19 in hospital settings, except for smoking. We provide evidence of potential sociodemographic factors associated with a positive test, including deprivation, population density, ethnicity, and chronic kidney disease.

**Funding:**

Wellcome Trust.

## Introduction

The world is in the midst of a pandemic caused by severe acute respiratory syndrome coronavirus 2 (SARS-CoV-2), which causes COVID-19.^1^ In the UK, the first cases were detected in late January, 2020, and community transmission began at the end of that month.^2^ Initial reports from China, Italy, and Spain described clinical characteristics of people diagnosed with COVID-19 and risk factors for poor outcomes, which include older age, male sex, cardiovascular disease, hypertension, and diabetes.^3^, ^4^ However, most research to date has been done among patients admitted to hospital with COVID-19, meaning risk factors for infection in the general population cannot be directly assessed. Use of primary care data could help identify risk factors for SARS-CoV-2 infection to inform patient management, public health measures, and more personalised advice to patient groups.^5^

**Research in context**

**Evidence before this study**

We searched PubMed, MEDLINE, and Trip Medical Database from inception to April 14, 2020, for community-based studies that described the epidemiology of severe acute respiratory syndrome coronavirus 2 (SARS-CoV-2) or the associated illness, COVID-19, using the terms “(COVID-19 or 2019-nCoV or SARS-CoV-2) AND (primary care or general practice or family practice or community)”, with no language restrictions. We found no relevant studies. Hospital-based studies have reported increasing age, male sex, and certain comorbidities, such as hypertension and diabetes, to be associated with more severe COVID-19 disease. Whether these risk factors apply to the risk of SARS-CoV-2 infection in primary care is unclear.

**Added value of this study**

We did a cross-sectional study of patients with a SARS-CoV-2 test code result in the Oxford Royal College of General Practitioners Research and Surveillance Centre network between Jan 28 and April 4, 2020. We observed 587 patients with positive results and 3215 with negative results. Since we have sociodemographic and clinical data on patients in our sample, we could assess risk factors for a positive SARS-CoV-2 result, adjusted for potential confounding variables. Increasing age, male sex, population density, more deprived areas, and black ethnicity were associated with an increased risk of a positive SARS-CoV-2 test. Chronic kidney disease and obesity were the only clinical factors associated with a positive test. Current smokers had lower odds of a positive test. To our knowledge, this study is one of the first to investigate risk factors for testing positive for SARS-CoV-2 in the community.

**Implications of all the available evidence**

Our findings suggest some risk factors for SARS-CoV-2 infection in this primary care study are similar to those associated with more severe COVID-19 disease, with men and people older than 40 years at increased risk. Research is needed into the effect of chronic conditions on the risk of infection and disease severity, ethnic variations in COVID-19 incidence, and the risk to smokers.

The Oxford Royal College of General Practitioners (RCGP) Research and Surveillance Centre programme is one of the longest established primary care sentinel networks globally. It includes more than 500 urban and non-urban participating general practices, covering a population of over 4 million people (appendix p 1).^6^, ^7^, ^8^ The Oxford RCGP Research and Surveillance Network supports Public Health England in national surveillance of communicable diseases such as influenza^9^ and assessing vaccine effectiveness,^10^, ^11^ including during the 2009 influenza pandemic.^12^ The network has adapted for COVID-19 surveillance by enlarging approximately three-fold to improve coverage and by introducing self-swabbing at home to reduce the risk of disease transmission.^13^, ^14^ We aimed to identify demographic and clinical risk factors for testing positive for SARS-CoV-2 within this primary care surveillance programme.

## Methods

### Study design and participants

We did a cross-sectional study in patients in the Oxford RCGP Research and Surveillance Centre network who were tested for SARS-CoV-2 between Jan 28 and April 4, 2020. Pseudonymised SARS-CoV-2 results and other clinical and sociodemographic data were extracted from computerised primary care medical records of sentinel practices. These data allow estimation of household size,^15^ deprivation level, and rural–urban classification.^16^ Since the last week of January, 2020, Research and Surveillance Centre practices have submitted nasopharyngeal swabs to Public Health England for SARS-CoV-2 testing from patients presenting with symptoms of influenza or respiratory infections. We included tests done through Public Health England surveillance, contact tracing, and routine UK National Health Service (NHS) primary and secondary care services. Although Public Health England surveillance testing has continued largely unchanged throughout the study period, NHS testing initially focused on people who travelled to high-risk countries or close contact tracing, but it has more recently focused on hospital testing and testing of health-care workers.

RT-PCR testing for SARS-CoV-2 was done at the Public Health England Colindale Laboratory (London, UK) using previously described methods.^17^ From early March, testing from routine NHS services was also done in NHS laboratories using standardised, national quality-assured procedures.^18^ The analytical specificity of RT-PCR assays for SARS-CoV-2 is greater than 95% and the analytical sensitivity of tests is typically 90–95%, with comparable performance between commercial tests used in the NHS and those used in the Public Health England Colindale Laboratory. Because of the operational nature of this in-pandemic study, various sampling and diagnostic test arrangements were used, with associated quality-assurance procedures.

We included patients who were registered at an RCGP Research and Surveillance Centre practice on Sept 30, 2019, who had an entry in their medical record reporting a positive or negative test for SARS-CoV-2. We have developed a COVID-19 surveillance ontology to ensure consistency of case definition and included only people with a coded positive or negative test, and not those with suspected disease (appendix p 2).^19^ Patients with codes in their medical records suggesting they had declined any form of data sharing were excluded (around 2·2% of the registered population).

The data used for the analysis were pseudonymised at the point of extraction and encrypted before uploading to the Clinical Informatics Research Group secure server. Personal data were not identifiable during the analysis. The data extraction was done as part of national surveillance work commissioned by Public Health England and approved under Regulation 3 of The Health Service (Control of Patient Information) Regulations 2002.^20^ This study was approved by the RCGP Research and Surveillance Centre study approval committee and was classified as a study of usual practice.^21^ Therefore, no further ethical approval was required.

### Study variables

We included the following independent demographic variables: age, sex, and ethnicity, using an ontology to maximise case identification;^22^ practice-level deprivation using the English Index of Multiple Deprivation quintiles (we combined the two most deprived quintiles as there was a low frequency of testing, leading to sparse data, in the most deprived quintile);^23^ household size based on pseudonymised patient address; and rural–urban classification. We included the latest recording of the following clinical variables, which are similar to those associated with increased susceptibility to influenza: body-mass index (BMI); smoking status; pregnancy; hypertension; chronic kidney disease; coronary heart disease; chronic respiratory disease, including asthma and chronic obstructive pulmonary disease; and type 1 and type 2 diabetes. We created a variable combining patients with malignancy and immunocompromise because there were small numbers in each group. Malignancy was identified using most recently recorded disease codes, and we used records of prescriptions for prednisolone and prescriptions for disease-modifying anti-rheumatic drugs as surrogates for immunosuppression. The outcome variable was testing positive for SARS-CoV-2.

### Statistical analysis

We used descriptive statistics and reported counts and proportions for categorical data and measures of distribution for continuous data. We described the proportion of participants with missing data for each variable (table 1). We tested for associations between individual covariates and the outcome of a positive test using univariate logistic regression models. We used multivariate logistic regression to identify variables that were associated with a positive test for SARS-CoV-2 after multiple imputation of missing values. We included all variables in the multivariable model. We imputed missing data using the multiple imputation by chained equations method, with five imputed datasets and ten iterations.^25^ For each variable, we specified a predictive mean matching model. We used all variables in the multivariable analysis and did not use auxiliary variables. All analysis results were aggregated with Rubin's rule after appropriate transformation.^26^ We checked the acceptability of the imputations by comparison of plots of the distribution of recorded and imputed values for all measurements. We used this method under the assumption that the missing observations for covariates were missing at random. We checked collinearity by measuring the variance inflationary factor for each covariate—all were deemed within acceptable bounds, with the maximum value less than 2·0. We also did sensitivity analyses using complete cases only and with missing ethnicity observations imputed using census data.^27^ In this analysis, for each person with missing ethnicity in a given lower super output area,^28^ we randomly assigned an ethnic group, matching the proportions of the ethnic group based on census proportions.

**Table 1:** Demographic and clinical characteristics of cohort

	**Participants (n=3802)**
**SARS-CoV-2 test result**	
Negative	3215 (84·6%)
Positive	587 (15·4%)
Missing	0
**Age (years)**	
0–17	499 (13·1%)
18–39	666 (17·5%)
40–64	1316 (34·6%)
65–74	557 (14·7%)
≥75	764 (20·1%)
Missing	0
**Sex**	
Female	2190 (57·6%)
Male	1612 (42·4%)
Missing	0
**Ethnicity**	
White	2497 (65·7%)
Asian	152 (4·0%)
Black	58 (1·5%)
Mixed, other	81 (2·1%)
Missing	1014 (26·7%)
Socioeconomic deprivation level*	
5 (least deprived)	1855 (48·8%)
4	633 (16·6%)
3	646 (17·0%)
1 and 2 (most deprived)	668 (17·6%)
Missing	0
**Household size**	
1	824 (21·7%)
2–4	2341 (61·6%)
5–8	408 (10·7%)
≥9	135 (3·6%)
Missing	94 (2·5%)
**Settlement or population density**	
Rural	1986 (52·2%)
Urban	1816 (47·8%)
Missing	0
**Smoking status**	
Non-smoker	1125 (29·6%)
Active smoker	413 (10·9%)
Ex-smoker	1753 (46·1%)
Missing	511 (13·4%)
**Pregnancy**	
No	3742 (98·4%)
Yes	60 (1·6%)
Missing	0
**BMI**†	
Normal weight	1296 (34·1%)
Overweight	1095 (28·8%)
Obese	680 (17·9%)
Severely obese	145 (3·8%)
Missing	586 (15·4%)
**Hypertension**	
No	2708 (71·2%)
Yes	1094 (28·8%)
Missing	0
**Chronic kidney disease**	
No	3595 (94·6%)
Yes	207 (5·4%)
Missing	0
**Diabetes**	
No	3299 (86·8)
Yes	503 (13·2)
Missing	0
**Chronic heart disease**	
No	3202 (84·2%)
Yes	600 (15·8%)
Missing	0
**Chronic respiratory disease**	
No	3544 (93·2%)
Yes	258 (6·8%)
Missing	0
**Malignancy or immunocompromised**	
No	3164 (83·2%)
Yes	638 (16·8%)
Missing	0

Data are n (%). SARS-CoV-2=severe acute respiratory syndrome coronavirus 2. BMI=body-mass index.

* Socioeconomic deprivation level was assessed at the practice level using the English Index of Multiple Deprivation quintiles.^23^

† BMI categories were based on WHO classification^24^ (normal weight 18·5–24·9 kg/m^2^, overweight 25·0–29·9 kg/m^2^, obese 30·0–39·9 kg/m^2^, severely obese ≥40 kg/m^2^).

We used R version 3.5.3 for all analyses; we used the R library mice 3.4.0 for the multiple imputation routine.

### Role of the funding source

The funder of the study had no role in study design, data collection, data analysis, data interpretation, or writing of the paper. The corresponding author had full access to all the data in the study and had final responsibility for the decision to submit for publication.

## Results

Between Jan 28 and April 4, 2020, we observed 587 patients with positive SARS-CoV-2 results and 3215 with negative results in the surveillance programme. The first positive case presented on Jan 30, 2020, and 100 cases was reached on March 17, 2020. Overall, 2190 (57·6%) of 3802 patients were female and 2497 (65·7%) were white (table 1). The median age of patients who had a test was 58·0 years (IQR 34–73) for men and 51·5 years (33–70) for women. 1986 (52·2%) patients lived in rural areas, and 1855 (48·8%) were ranked as least deprived (quintile 5) according to the Index of Multiple Deprivation. The most common clinical conditions were hypertension (1094 [28·8%] patients) and chronic heart disease (600 [15·8%] patients). 267 (7%) results were obtained from Public Health England surveillance testing, whereas 3535 (93%) were identified through surveillance of primary care medical records.

In univariable analysis, the odds of testing positive for SARS-CoV-2 were higher among older people, men, and people of ethnicity other than white, and people living in more deprived areas (table 2). The odds of a positive test were lower in households with two to four or five to eight people. Among clinical factors in the univariable analysis, chronic kidney disease, obesity, malignancy or immunocompromised, diabetes, chronic respiratory disease, chronic heart disease, and hypertension were all associated with increased odds of a positive test for SARS-CoV-2 (table 2). Active smoking was associated with decreased odds of a positive test.

**Table 2:** Univariable analysis of demographic and clinical risk factors for testing positive for SARS-CoV-2

		**SARS-CoV-2 positivity**	**Unadjusted odds ratio (95% CI)**	**p value**
Age (years)		..	..	<0·0001
	0–17	23/499 (4·6%)	1 (ref)	..
	18–39	84/666 (12·6%)	2·98 (1·85–4·81)	..
	40–64	243/1316 (18·5%)	4·69 (3·00–7·28)	..
	65–74	88/557 (15·8%)	3·88 (2·40–6·25)	..
	≥75	149/764 (19·5%)	5·00 (3·18–7·90)	..
Sex		..	..	<0·0001
	Female	291/2190 (13·3%)	1 (ref)	..
	Male	296/1612 (18·4%)	1·47 (1·23–1·75)	..
Ethnicity		..	..	<0·0001
	White	388/2497 (15·5%)	1 (ref)	..
	Asian	47/152 (30·9%)	2·43 (1·70–3·49)	..
	Black	36/58 (62·1%)	8·90 (5·20–15·30)	..
	Mixed, other	20/81 (24·7%)	1·78 (1·10–2·90)	..
	Missing	96/1014 (9·5%)	0·57 (0·45–0·72)	..
Socioeconomic deprivation level*		..	..	<0·0001
	5 (least deprived)	143/1855 (7·7%)	1·00 (ref)	..
	4	112/633 (17·7%)	2·58 (1·97–3·36)	..
	3	135/646 (20·9%)	3·16 (2·45–4·10)	..
	1 and 2 (most deprived)	197/668 (29·5%)	5·01 (3·95–6·35)	..
Household size		..	..	<0·0001
	1	163/824 (19·8%)	1·00 (ref)	..
	2–4	320/2341 (13·7%)	0·64 (0·52–0·79)	..
	5–8	53/408 (13·0%)	0·61 (0·43–0·85)	..
	≥9	35/135 (25·9%)	1·42 (0·93–2·16)	..
	Missing	16/94 (17·0%)	0·83 (0·47–1·46)	..
Settlement or population density				<0·0001
	Rural	111/1986 (5·6%)	1 (ref)	..
	Urban	476/1816 (26·2%)	6·00 (4·82–7·46)	..
Smoking status		..	..	<0·0001
	Non-smoker	201/1125 (17·9%)	1 (ref)	..
	Active smoker	47/413 (11·4%)	0·59 (0·42–0·83)	..
	Ex-smoker	303/1753 (17·3%)	0·96 (0·79–1·17)	..
	Missing	36/511 (7·0%)	0·35 (0·24–0·51)	..
Pregnancy		..	..	0·0400
	No	583/3742 (15·6%)	1 (ref)	..
	Yes	4/60 (6·7%)	0·39 (0·14–1·10)	..
BMI†		..	..	<0·0001
	Normal weight	171/1296 (13·2%)	1 (ref)	..
	Overweight	198/1095 (18·1%)	1·45 (1·20–1·80)	..
	Obese	142/680 (20·9%)	1·74 (1·36–2·20)	..
	Severely obese	26/145 (17·9%)	1·44 (0·91–2·27)	..
	Missing	50/586 (8·5%)	0·61 (0·44–0·85)	..
Hypertension		..	..	<0·0001
	No	378/2708 (14·0%)	1 (ref)	..
	Yes	209/1094 (19·1%)	1·46 (1·20–1·75)	..
Chronic kidney disease		..	..	<0·0001
	No	519/3595 (14·4%)	1 (ref)	..
	Yes	68/207 (32·9%)	2·90 (2·14–3·93)	..
Diabetes		..	..	<0·0001
	No	473/3299 (14·3%)	1 (ref)	..
	Yes	114/503 (22·7%)	1·75 (1·40–2·20)	..
Chronic heart disease		..	..	<0·0001
	No	451/3202 (14·1%)	1 (ref)	..
	Yes	136/600 (22·7%)	1·79 (1·44–2·20)	..
Chronic respiratory disease		..	..	<0·0001
	No	529/3544 (14·9%)	1 (ref)	..
	Yes	58/258 (22·5%)	1·65 (1·21–2·25)	..
Malignancy or immunocompromised		..	..	0·0010
	No	460/3164 (14·5%)	1 (ref)	..
	Yes	127/638 (19·9%)	1·46 (1·17–1·82)	..

Data are n/N (%), unless otherwise indicated. SARS-CoV-2=severe acute respiratory syndrome coronavirus 2. BMI=body-mass index.

* Socioeconomic deprivation level was assessed at the practice level using the English Index of Multiple Deprivation quintiles.^23^

† BMI categories were based on WHO classification^24^ (normal weight 18·5–24·9 kg/m^2^, overweight 25·0–29·9 kg/m^2^, obese 30·0–39·9 kg/m^2^, severely obese ≥40 kg/m^2^).

In multivariable analysis, adjusted for all other variables in table 3, male sex remained independently associated with testing positive for SARS-CoV-2 (adjusted odds ratio [OR] 1·55, 95% CI 1·27–1·89). Adults were at increased risk compared with children, and people aged 40–64 years (5·36, 3·28–8·76) and 75 years and older (5·23, 3·00–9·09) were at greatest risk. Compared with white people, black people remained at increased risk of testing positive for SARS-CoV-2 (4·75, 2·65–8·51). Urban areas (4·59, 3·57–5·90) versus rural areas, and more deprived areas (most deprived *vs* least deprived; 2·03, 1·51–2·71) were associated with increased odds of a positive SARS-CoV-2 test.

**Table 3:** Multivariable analysis of risk factors for testing positive for SARS-CoV-2

		**Adjusted odds ratio (95% CI)**	**p value**
Age (years)		..	<0·0001
	0–17	1 (ref)	..
	18–39	2·83 (1·69–4·74)	..
	40–64	5·36 (3·28–8·76)	..
	65–74	4·41 (2·52–7·69)	..
	≥75	5·23 (3·00–9·09)	..
Sex		..	<0·0001
	Female	1 (ref)	..
	Male	1·55 (1·27–1·89)	..
Ethnicity		..	<0·0001
	White	1 (ref)	..
	Asian	1·46 (0·94–2·29)	..
	Black	4·75 (2·65–8·51)	..
	Mixed, other	1·71 (0·97–3·01)	..
Socioeconomic deprivation level*		..	<0·0001
	5 (least deprived)	1 (ref)	..
	4	1·51 (1·13–2·03)	..
	3	2·35 (1·78–3·11)	..
	1 and 2 (most deprived)	2·03 (1·51–2·71)	..
Household size		..	0·4900
	1	1 (ref)	..
	2–4	0·97 (0·77–1·23)	..
	5–8	0·86 (0·57–1·31)	..
	≥9	1·29 (0·80–2·07)	..
Settlement or population density		..	<0·0001
	Rural	1 (ref)	..
	Urban	4·59 (3·57–5·90)	..
Smoking status		..	0·0010
	Non-smoker	1 (ref)	..
	Active smoker	0·49 (0·34–0·71)	..
	Ex-smoker	0·87 (0·69–1·10)	..
BMI†		..	0·0090
	Normal weight	1 (ref)	..
	Overweight	1·26 (0·99–1·61)	..
	Obese	1·41 (1·04–1·91)	..
	Severely obese	1·28 (0·78–2·10)	..
Hypertension		..	0·3100
	No	1 (ref)	..
	Yes	0·89 (0·69–1·14)	..
Chronic kidney disease		..	<0·0001
	No	1 (ref)	..
	Yes	1·91 (1·31–2·78)	..
Diabetes		..	0·8300
	No	1 (ref)	..
	Yes	1·03 (0·78–1·36)	..
Chronic heart disease		..	0·1800
	No	1 (ref)	..
	Yes	1·21 (0·92–1·60)	..
Chronic respiratory disease		..	0·8200
	No	1 (ref)	..
	Yes	1·04 (0·72–1·50)	..
Malignancy or immunocompromised		..	0·9800
	No	1 (ref)	..
	Yes	1·01 (0·78–1·31)	..

SARS-CoV-2=severe acute respiratory syndrome coronavirus 2. BMI=body-mass index.

* Socioeconomic deprivation level was assessed at the practice level using the English Index of Multiple Deprivation quintiles.^23^

† BMI categories were based on WHO classification^24^ (normal weight 18·5–24·9 kg/m^2^, overweight 25·0–29·9 kg/m^2^, obese 30·0–39·9 kg/m^2^, severely obese ≥40 kg/m^2^).

Active smoking was associated with decreased odds of a positive SARS-CoV-2 test result (adjusted OR 0·49, 95% CI 0·34–0·71). People with chronic kidney disease were more likely than those without to test positive for SARS-CoV-2 in the adjusted analysis (1·91, 1·31–2·78), but there was no significant association with the other chronic conditions (table 3). We found evidence of increased odds of a positive test among people with obesity compared to those of normal weight (1·41, 1·04–1·91).

In sensitivity analyses, we did a complete case analysis (appendix p 3) and imputed missing ethnicity data using local census data (appendix p 4), but found no marked differences in our results.

## Discussion

We report one of the first and largest cross-sectional analyses using primary care data to assess risk factors for testing positive for SARS-CoV-2. In our sample, we found increasing age, male sex, increasing deprivation, urban location, and black ethnicity were associated with increased odds of a positive SARS-CoV-2 test. Current smoking was linked with decreased odds of a positive test. Chronic kidney disease and increased BMI were the only clinical factors independently associated with a positive test.

A literature review suggested that COVID-19 has affected more men than women, and principally those aged 30–65 years, with around half of cases being older than 50 years.^29^ We found a similar increased risk of a positive SARS-CoV-2 test in men, and in people older than 40 years.

SARS-CoV-2 transmission is known to be associated with high population density due to increased social mixing,^30^ which is consistent with our finding of higher odds of a positive test in urban areas. Social deprivation has been associated with increased risk of other respiratory infections,^31^ and there is evidence that the risk of COVID-19-related death is higher in more deprived parts of England, although this analysis has not been adjusted for potential confounders.^32^ We found an association between increasing deprivation and increased odds of a positive test, independent of household size, urban location, and smoking. Perhaps surprisingly, we did not find an association between increased household size and risk of SARS-CoV-2 positivity, despite a previously reported higher risk of transmission among household contacts.^33^ Behavioural responses to social distancing measures might have accounted for this finding. For example, small households could be studio flats or single-room occupancies without communal space, such that people might be more inclined to risk infection by leaving home.

Preliminary evidence has raised concerns regarding the potential increased risk of adverse COVID-19 outcomes among people of Asian and black ethnicities, but few epidemiological studies have assessed risk by ethnic group.^34^ An analysis of 3370 people admitted to intensive care in the UK with confirmed COVID-19 found that 402 (11·9%) were black, 486 (14·4%) were Asian, and 2236 (66·4%) were white,^35^ compared with respective national figures of 3·3%, 7·5%, and 86·0%.^36^ These results did not adjust for potential sociodemographic or clinical confounders. Overall numbers of black people, Asian people, and people from minority ethnic groups were small in our study, meaning our results should be interpreted with caution. However, we found that black people had higher odds of a positive SARS-CoV-2 test result than white people, which remained significant after adjusting for comorbidities such as hypertension and diabetes, the prevalence of which is increased in black ethnic groups.^37^ Other socioeconomic factors that we did not measure, such as employment in high-risk positions, education, income, and structural barriers to health care, might have contributed to this association and should be urgently explored.

Systematic reviews have shown that people with COVID-19 who have chronic comorbidities such as hypertension, diabetes, and cardiovascular disease are at high risk of progressing to severe COVID-19 disease.^38^ Risk factors for SARS-CoV-2 infection could be different, and we found no evidence of an association between these conditions and a positive SARS-CoV-2 test. We found that chronic kidney disease and obesity were associated with testing positive for SARS-CoV-2. Both chronic kidney disease and obesity have been associated with increased risk of other respiratory infections.^39^, ^40^, ^41^ Angiotensin-converting enzyme inhibitors are recommended treatments for chronic kidney disease and have been postulated to impact SARS-CoV-2 host-cell interactions.^42^ However observational evidence does not support this effect,^43^, ^44^, ^45^ and further analyses to investigate the relationship between medications, chronic illnesses, and SARS-CoV-2 positivity.

Previous studies have reported that smoking is associated with increased risk of intensive care unit admission or death among people with COVID-19.^46^ However, several studies reported a low prevalence of smoking among people with COVID-19. A Chinese study found that only 137 (12·6%) of 1085 patients with COVID-19 were current smokers, compared with 27·7% of Chinese adults,^47^ and an analysis of cases by the US Centers for Disease Control and Prevention found only 96 (1·3%) of 7162 COVID-19 cases were active smokers, compared with 13·7% in the general US population.^48^ These studies could be biased by confounding and by difficulties in accurately identifying current smokers among patients unwell with COVID-19. We found that active smoking was associated with lower odds of having a positive test result. There are several plausible reasons for this result. Active smoking might affect nasopharyngeal viral load and therefore affect RT-PCR test sensitivity, rather than protecting against actual infection, although this effect is not known to occur with influenza RT-PCR testing.^49^ Alternatively, as patients with symptoms are more likely to have been tested and included in our analysis, selection bias could affect this result.^50^ Smokers are more likely to have a cough, meaning they might also be more likely to be tested for SARS-CoV-2 than non-smokers, even if they are SARS-CoV-2 negative. This more frequent testing could increase the proportion of smokers with negative SARS-CoV-2 results in our sample, which would bias our results. However, the proportion of smokers in our study was low. Furthermore, ex-smokers and people with chronic lung disease would also be expected to cough more, but these groups did not have higher odds of SARS-CoV-2 test positivity. Therefore, the relationship between smoking and SARS-CoV-2 infection merits further investigation. Nicotine might downregulate angiotensin-converting enzyme 2 receptors,^51^ which are used by SARS-CoV-2 for cell entry, although studies have found increased angiotensin-converting enzyme 2 lung expression among smokers and people with chronic obstructive pulmonary disease.^52^, ^53^ Our findings should not be used to conclude that smoking prevents SARS-CoV-2 infection, or to encourage ongoing smoking, particularly given the well documented harms to overall health from smoking, the potential for smoking to increase COVID-19 disease severity,^46^ and the possible alternative explanations for these findings.

To our knowledge, our study is one of the first to report risk factors for testing positive for SARS-CoV-2. Our use of rich primary care surveillance data allowed adjustment for potential confounding factors. The Oxford RCGP Research and Surveillance Centre is an established network of sentinel practices, meaning clinicians are experienced in undertaking surveillance research and use coding ontologies to standardise reporting.

Like all routine datasets, some data will be missing from our set. Where data are missing at random, multiple imputation has the potential to reduce bias and improve precision. However, the missing at random assumption is not testable. In certain situations when the missing at random assumption does not hold, we can rely on a complete-case analysis to provide unbiased estimates (eg, when the likelihood of being a complete case is independent of the outcome, conditional on the other covariates).^54^ In this study, we presented both approaches, with similar results. We acknowledge that ethnicity, for example, might not be missing at random. However, our findings remained unchanged in a sensitivity analysis that did not rely on the missing at random assumption, in which we imputed missing ethnicity based on ethnicity proportions in each participant's local geographical area.

Although our study population of primary care patients is likely to be more similar to the general population than that of hospital-based studies, there remains a risk of selection bias because results might reflect the groups of patients who were more likely to present for assessment and be selected for SARS-CoV-2 testing in accordance with guidelines. If certain groups (eg, men, people in deprived areas, non-smokers, and black people) are only likely to present or be tested when more severely unwell, those who were tested could be more likely to be positive for COVID-19. Conversely, groups with lower thresholds for presentation might be tested with less severe symptoms, and therefore be more likely to test negative. It was not possible to assess the effects of thresholds for presentation and changes in testing guidelines in this analysis. Population-based surveys should ensure consistent levels of testing across subgroups as far as possible to reduce the risk of selection bias.

Although RT-PCR testing is the gold standard for SARS-CoV-2 diagnosis, overall test sensitivity in clinical use might be reduced by factors such as swab technique and the timing relative to symptom onset. Therefore, some SARS-CoV-2 cases could have been missed, particularly among patients with lower viral loads, which could bias results if any of the variables that we studied (eg, active smoking) were associated with differences in viral load, rather than actual infection. Also, the sentinel network changed from in-practice nasopharyngeal swabbing to self-swabbing on March 14, 2020, which nonetheless has been found to be a reliable method when testing for influenza.^55^

Further data are needed to establish the epidemiology of SARS-CoV-2, particularly in relation to emerging factors such as ethnicity, deprivation, population density, and smoking. Population-based surveys could help reduce selection bias and ensure adequate inclusion of different population subgroups. Our data from primary care could help monitor incident infections and, therefore, the effect of public health mearues, and we plan analyses of rates of hospitalisation and death as the pandemic unfolds.

In conclusion, primary care sentinel network data provide important insights into the epidemiology of SARS-CoV-2, although our study is limited by its small scale and selection of patients presenting for SARS-CoV-2 testing through routing health-care services. Our findings on smoking might be due to presentation confounding and should not encourage people to continue or take up smoking. Increasing age, male sex, socioeconomic deprivation, increased population density, black ethnicity, chronic kidney disease, and obesity were all associated with increased risk of a positive SARS-CoV-2 test.

## Data sharing

The Royal College of General Practitioners (RCGP) Research and Surveillance Centre dataset can be accessed by researchers. Approval is on a project-by-project basis. Ethical approval by a UK National Health Service Research Ethics Committee is needed before any data release or other appropriate approval. Researchers wishing to directly analyse patient-level pseudonymised data will be required to complete information governance training and work on the data from the secure servers at the University of Surrey (Guildford, UK). Patient-level data cannot be taken out of the secure network. We encourage interested researchers to attend the short courses on how to analyse primary care or RCGP Research and Surveillance Centre data, which are open to enrolment twice a year.
