# Supplementary material for: Risk factors for SARS-CoV-2 among patients in the Oxford Royal College of General Practitioners Research and Surveillance Centre primary care network: a cross-sectional study
Source: Lancet Infect Dis. 2020 Sep;20(9):1034–42. doi: 10.1016/S1473-3099(20)30371-6 (PMC7228715; doi:10.1016/S1473-3099(20)30371-6)
Supplement: Supplementary appendix [file mmc1.pdf]

THE LANCET
Infectious Diseases

Supplementary appendix

This appendix formed part of the original submission and has been peer reviewed. We post it as supplied by the authors.

Supplement to: de Lusignan S, Dorward J, Correa A, et al. Risk factors for SARS-CoV-2 among patients in the Oxford Royal College of General Practitioners Research and Surveillance Centre primary care network: a cross-sectional study. *Lancet Infect Dis* 2020; published online May 15. http://dx.doi.org/10.1016/S1473-3099(20)30371-6.

Supplementary material

Figure S1: Ox-RCGP RSC practices by NHS Region in England

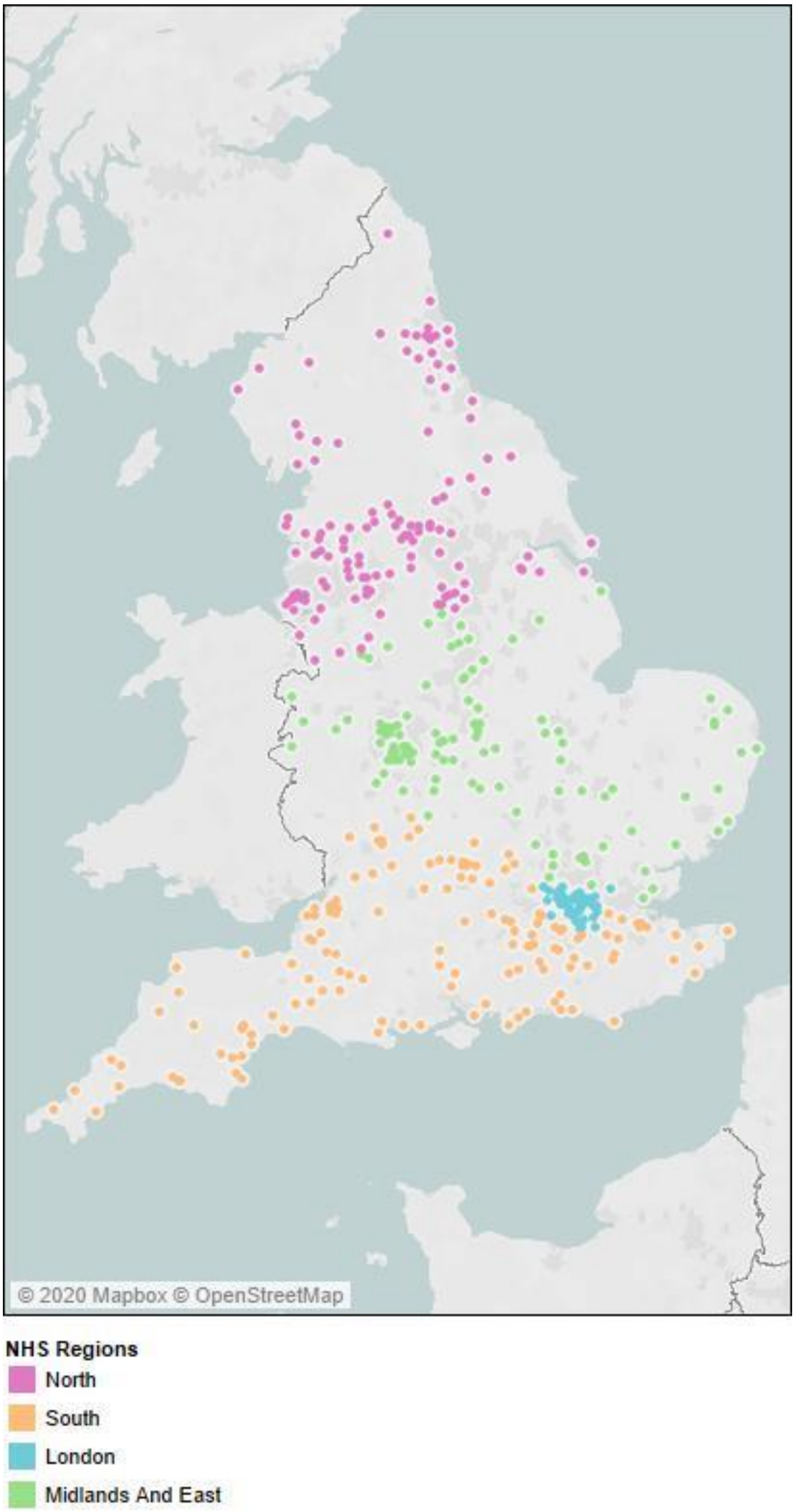

Created using www.openstreetmap.org, licensed under CC BY-SA. Data is available under the Open Database Licence.

Table S1: Codes used to extract SARS-CoV-2 test results from electronic health records

Concept Type	Code	Term
Positive	1240521000000100	Otitis media caused by 2019 novel coronavirus (disorder)
Positive	1240531000000100	Myocarditis caused by 2019 novel coronavirus (disorder)
Positive	1240541000000100	Infection of upper respiratory tract caused by 2019 novel coronavirus (disorder)
Positive	1240551000000100	Pneumonia caused by 2019 novel coronavirus (disorder)
Positive	1240561000000100	Encephalopathy caused by 2019 novel coronavirus (disorder)
Positive	1240571000000100	Gastroenteritis caused by 2019 novel coronavirus (disorder)
Positive	1240581000000100	2019 novel coronavirus detected (finding)
Positive	1240751000000100	Disease caused by 2019 novel coronavirus (disorder)
Positive	186747009	Coronavirus infection (disorder)
Negative	1240591000000100	2019 novel coronavirus not detected (finding)
Positive	EMISNQCO303	Confirmed 2019-nCoV (novel coronavirus) infection
Positive	Y20d1	Severe acute respiratory syndrome coronavirus 2 detected (finding)
Positive	Y210b	Infection of upper respiratory tract caused by severe acute respiratory syndrome coronavirus 2 (disorder)
Negative	EMISNQEX59	Excluded 2019-nCoV (novel coronavirus) infection
Negative	Y20d2	Excluded 2019-nCoV (Wuhan) infection

Table S2: Sensitivity analysis of complete cases using multivariate logistic regression (N=2385)

Variable	Level	OR (95% CI)	P-value
Age (years)	*0-17*	*1*	
	18-39	*3.25 (0.73-14.41)*	*0.1212*
	40-64	*5.56 (1.27-24.36)*	*0.0229*
	65-74	*4.90 (1.09-22.10)*	*0.0384*
	75+	*5.45 (1.21-24.63)*	*0.0274*
Sex	*Female*	*1*	
	Male	*1.52 (1.20-1.93)*	*0.0005*
Ethnicity	*White*	*1*	
	Asian	*1.31 (0.85-2.00)*	*0.2230*
	Black	*5.52 (2.75-11.06)*	*<0.0001*
	Mixed, Other	*1.55 (0.84-2.86)*	*0.1592*
Socioeconomic deprivation level - Index of Multiple Deprivation	*5 (Least deprived)*	*1*	
	4	*1.47 (1.04-2.10)*	*0.0311*
	3	*2.65 (1.92-3.66)*	*<0.0001*
	1 & 2 (Most Deprived)	*2.19 (1.56-3.07)*	*<0.0001*
Settlement / Population Density	*Rural*	*1*	
	Urban	*4.75 (3.55-6.36)*	*<0.0001*
Smoking Status	*Non-smoker*	*1*	
	Active Smoker	*0.40 (0.26-0.63)*	*0.0001*
	Ex-smoker	*0.94 (0.73-1.22)*	*0.6666*
*BMI**	*Normal Weight*	*1*	
	Over Weight	*1.25 (0.95-1.66)*	*0.1119*
	Obese	*1.61 (1.18-2.21)*	*0.0029*
	Severely Obese	*1.49 (0.85-2.59)*	*0.1633*
Hypertension	*No*	*1*	
	Yes	*0.99 (0.75-1.31)*	*0.9302*
CKD	*No*	*1*	
	Yes	*1.82 (1.22-2.71)*	*0.0033*
Diabetes	*No*	*1*	
	Yes	*0.99 (0.73-1.34)*	*0.9279*
Chronic Heart Disease	*No*	*1*	
	Yes	*1.14 (0.83-1.56)*	*0.4245*
Chronic Respiratory Disease	*No*	*1*	
	Yes	*1.06 (0.71-1.60)*	*0.7731*
Malignancy and Immuno-compromised	*No*	*1*	
	Yes	*0.92 (0.68-1.25)*	*0.5974*

*BMI categories based on World Health Organization classification[i] (normal weight 18.5-24.9 kg/m^2, overweight 25.0-29.9 kg/m^2, obese 30.0-39.9 kg/m^2, severely obese ≥40 kg/m^2)

Table S3: Sensitivity analysis with Ethnicity Group assigned at random in proportion to the local super output area ethnic group proportions (from census data).

**Variable**	**Level**	**OR (95% CI)**	**P-value**
Age (years)	*0-17*	*1*	
	18-39	*2.78 (1.65-4.67)*	*0.0001*
	40-64	*5.26 (3.20-8.64)*	*<0.0001*
	65-74	*4.24 (2.43-7.42)*	*<0.0001*
	75+	*4.99 (2.85-8.68)*	*<0.0001*
Sex	*Female*	*1*	
	Male	*1.54 (1.26-1.88)*	*<0.0001*
Ethnicity	*White*	*1*	
	Asian	*1.38 (0.95-2.03)*	*0.1000*
	Black	*4.99 (2.82-8.86)*	*<0.0001*
	Mixed, Other	*1.63 (0.96-2.75)*	*0.0683*
Socioeconomic deprivation level - Index of Multiple Deprivation	*5 (Least deprived)*	*1*	
	4	*1.50 (1.12-2.01)*	*0.0067*
	3	*2.34 (1.77-3.10)*	*<0.0001*
	1 & 2 (Most Deprived)	*2.10 (1.56-2.78)*	*<0.0001*
Settlement / Population Density	*Rural*	*1*	
	Urban	*4.62(3.60-5.92)*	*<0.0001*
Smoking Status	*Non-smoker*	*1*	
	Active Smoker	*0.47(0.32-0.68)*	*0.0001*
	Ex-smoker	*0.86(0.69-1.10)*	*0.2035*
*BMI**	*Normal Weight*	*1*	
	Over Weight	*1.25 (0.97-1.59)*	*0.0795*
	Obese	*1.39 (1.04-1.86)*	*0.0255*
	Severely Obese	*1.25 (0.75-2.10)*	*0.3964*
Hypertension	*No*	*1*	
	Yes	*0.90 (0.70-1.16)*	*0.4110*
CKD	*No*	*1*	
	Yes	*1.91 (1.31-2.79)*	*0.0008*
Diabetes	*No*	*1*	
	Yes	*1.0 (0.79-1.38)*	*0.7692*
Chronic Heart Disease	*No*	*1*	
	Yes	*1.22 (0.92-1.61)*	*0.1649*
Chronic Respiratory Disease	*No*	*1*	
	Yes	*1.0 (0.72-1.49)*	*0.8506*
Malignancy and Immuno-compromised	*No*	*1*	
	Yes	*1.0 (0.78-1.31)*	*0.9300*

*BMI categories based on World Health Organization classification[1] (normal weight 18.5-24.9 kg/m^2, overweight 25.0-29.9 kg/m^2, obese 30.0-39.9 kg/m^2, severely obese ≥40 kg/m^2)

[i] World Health Organization. Physical status: the use and interpretation of anthropometry. Report of a WHO Expert Committee. World Health Organ Tech Rep Ser. 1995;854:1-452. www.who.int/childgrowth/publications/physical_status/en/ Accessed 28/04/2020
